# Coronavirus accessory protein ORF3 biology and its contribution to viral behavior and pathogenesis

**DOI:** 10.1016/j.isci.2023.106280

**Published:** 2023-02-28

**Authors:** Fusheng Si, Shuai Song, Ruisong Yu, Zhen Li, Wenqiang Wei, Chao Wu

**Affiliations:** 1 Institute of Animal Science and Veterinary Medicine, Shanghai Academy of Agricultural Sciences, Shanghai Key Laboratory of Agricultural Genetics and Breeding, Shanghai Engineering Research Center of Breeding Pig, Shanghai 201106, P.R. China; 2 Institute of Animal Health, Guangdong Academy of Agricultural Sciences, Scientific Observation and Experiment Station of Veterinary Drugs and Diagnostic Techniques of Guangdong Province, Ministry of Agriculture of Rural Affairs, and Key Laboratory of Animal Disease Prevention of Guangdong Province, Guangzhou 510640, P.R. China; 3 Department of Microbiology, School of Basic Medical Sciences, Henan University, Kaifeng, Henan 475004, P.R. China; 4 Department of Pathology and Immunology, Washington University in St. Louis, St. Louis, MO 63110, USA

**Keywords:** Porcine molecular biology, Protein, Virology

## Abstract

Coronavirus porcine epidemic diarrhea virus (PEDV) is classified in the genus *Alphacoronavirus*, family *Coronaviridae* that encodes the only accessory protein, ORF3 protein. However, how ORF3 contributes to viral pathogenicity, adaptability, and replication is obscure. In this review, we summarize current knowledge and identify gaps in many aspects of ORF3 protein in PEDV, with emphasis on its unique biological features, including membrane topology, Golgi retention mechanism, potential intrinsic disordered property, functional motifs, protein glycosylation, and codon usage phenotypes related to genetic evolution and gene expression. In addition, we propose intriguing questions related to ORF3 protein that we hope to stimulate further studies and encourage collaboration among virologists worldwide to provide constructive knowledge about the unique characteristics and biological functions of ORF3 protein, by which their potential role in clarifying viral behavior and pathogenesis can be possible.

## Graphical abstract

**Figure:**
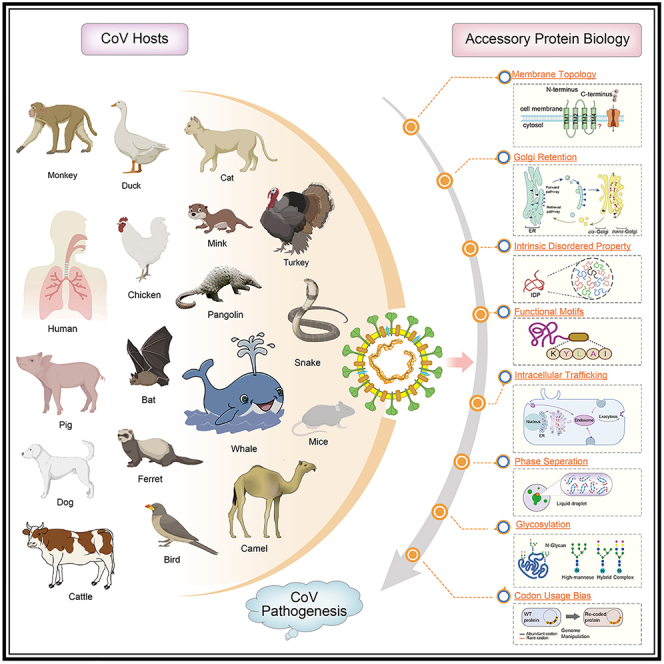

## Introduction

**Figure 1:**
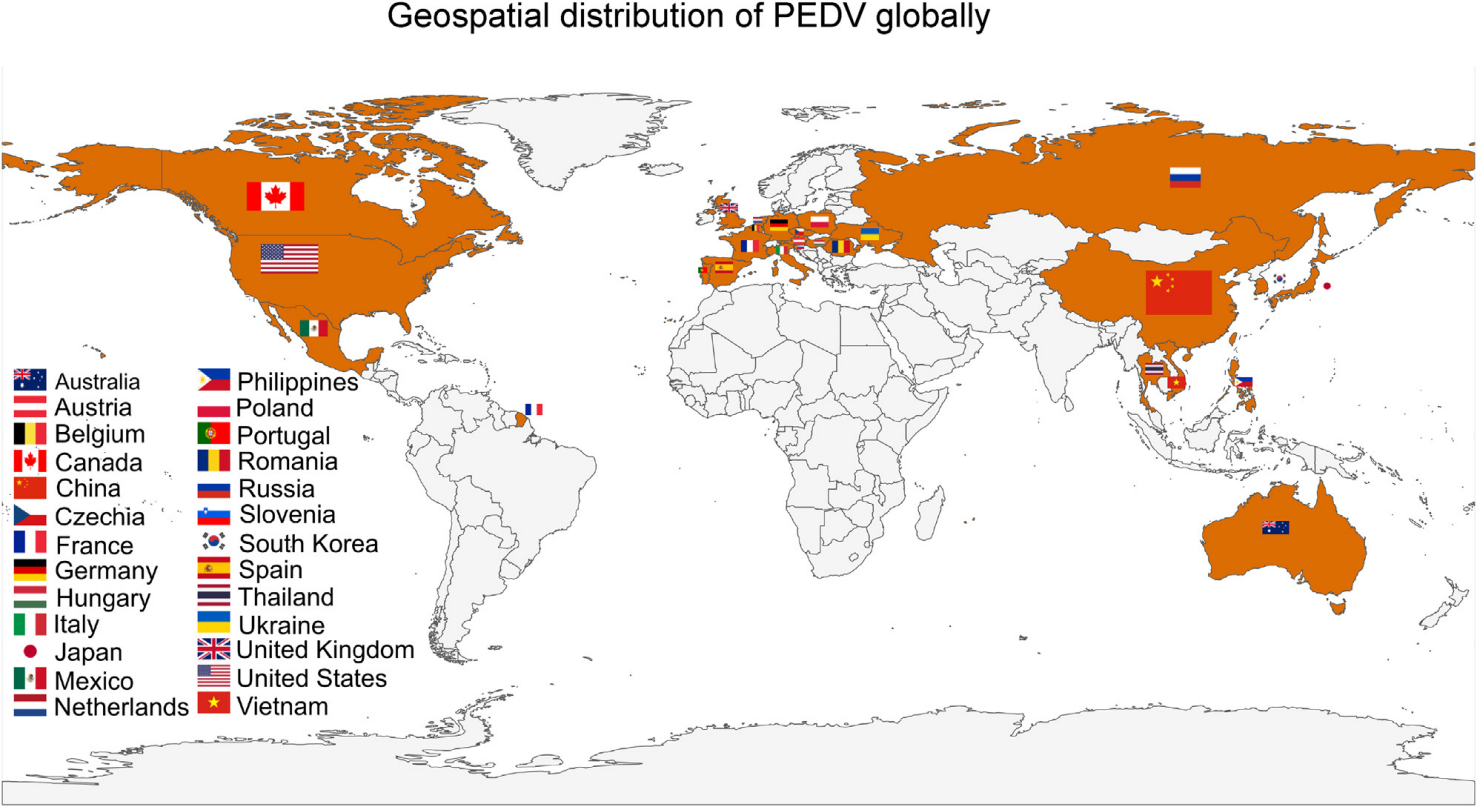
Geographic distribution of porcine epidemic diarrhea virus (PEDV) around the world The white areas mean no cases, whereas the orange marked regions indicate the high prevalence of PEDV infection. The map was visualized in RStudio using the packages natural earth, sf, and ggplot2.

Porcine epidemic diarrhea (PED) is an enteric disease characterized by acute diarrhea, vomiting, dehydration, and weight loss in pigs and has caused enormous economic losses in countries around the world.^1^ Although PED was first documented in the UK as early as 1971,^2^ the causing pathogen, PED virus (PEDV), was initially identified in Belgium in 1978.^3^ Subsequently, cases of PEDV infection were reported in several European countries, as well as Asian countries, such as China, Japan, South Korea, and Thailand (Figure 1).^4^ Since 2010, variants of PEDV associated with large-scale outbreaks of diarrhea have been documented in China. Suckling piglets afflicted by the severe pandemic suffered an 80–100% morbidity and a 50–90% mortality,^5^ which posed a serious threat to the Chinese pig industry. PEDV was first discovered in the USA in 2013.^6^ Since then, the virus has spread quickly across the country, killing many sick piglets and resulting in significant economic losses.^7^^,^^8^

Coronaviruses (CoVs) are a family of enveloped, positive-sense, and single-stranded RNA viruses that belong to the *Coronaviridae* family of order *Nidovirales* and can be divided into four genera, including *Alphacoronavirus* (α-CoV), *Betacoronavirus* (β-CoV), *Gammacoronavirus* (γ-CoV), and *Deltacoronavirus* (δ-CoV).^9^ CoVs are major threats to humans and vertebrate species. They can infect humans, livestock, birds, bats, mice, and many other wild animals, causing respiratory, enteric, hepatic, and neurological diseases.^10^ Taxonomically, PEDV is classified in the genus *Alphacoronavirus*, family *Coronaviridae*. Because of the lack of effective vaccines, PEDV remains one of the biggest risks to the swine industry on a global scale.^1^

The detailed molecular mechanism of PEDV invading cells, especially the interactions between virus protein and cellular receptor, is largely unknown. Porcine aminopeptidase N (pAPN), a 150-kDa glycosylated type II transmembrane (TM) protein partially expressed on the brush border membrane by porcine small intestinal villous enterocytes,^11^^,^^12^ was initially identified as the functional cellular receptor for PEDV.^13^^,^^14^ However, subsequent research found that the pAPN-deficient cell line, such as the African green monkey kidney (Vero) cell, is susceptible to PEDV infection,^13^^,^^15^^,^^16^ challenging pAPN’s role as a functional receptor. Indeed, some recent studies disputed the conventional wisdom regarding the crucial role played by pAPN during the PEDV infection and found that though pAPN promotes PEDV infectivity via aminopeptidase activity, it still was not an acknowledged cellular receptor of PEDV.^17^^,^^18^ Thus, the genuine virus receptor still awaits its identification, and robust evidence must be provided to stress this fundamental virological issue in elucidating the virus pathogenesis.

The coronavirus genome is distinct from other *Nidoviruses* because it encodes various accessory proteins in its 3’-proximal genomic regions, which appear important for viral pathogenesis but not necessary for virus replication. In the case of PEDV, ORF3 protein, which is the only accessory protein encoded by the PEDV genome, has not been studied as thoroughly as other viral structural proteins, and much of what is known about them comes from substantial functional investigations on severe acute respiratory syndrome coronavirus (SARS-CoV),^19^ severe acute respiratory syndrome coronavirus-2 (SARS-CoV-2),^20^ and other comparable viruses, such as Middle East respiratory syndrome coronavirus (MERS-CoV).^21^^,^^22^ Although ORF3 protein is not essential for PEDV replication, it plays an important role in cell cycle progression, stress responses, including apoptosis and autophagy, and innate immune responses. In addition, the evolutionary analysis indicated that the *ORF3* gene tends to be mutated and truncated during cell adaptation. Some novel field PEDV strains carrying naturally truncated *ORF3* gene were constantly emerging and closely related to viral pathogenesis.^23^^,^^24^ Therefore, extensive study of the biological function of ORF3 protein is important to further elucidate the pathogenic mechanism of PEDV.

Although a concise study about ORF3 accessory protein was reported with emphasis on the ORF3 and host interaction previously,^25^ there are still many unknown aspects regarding ORF3 protein’s biological characteristics, including the membrane topology, Golgi retention mechanism, potential intrinsic disordered property, functional motifs, protein post-translational modifications (PTMs), and codon usage phenotypes related to genetic evolution and gene expression. Given the important role of accessory protein in coronavirus infection and the research blind spots of PEDV accessory protein in many aspects, current knowledge of this PEDV only accessory protein, ORF3, is summarized with a focus on what is now known and which areas urgently require additional investigation.

## Virion properties and structure of the PEDV genome

**Figure 2:**
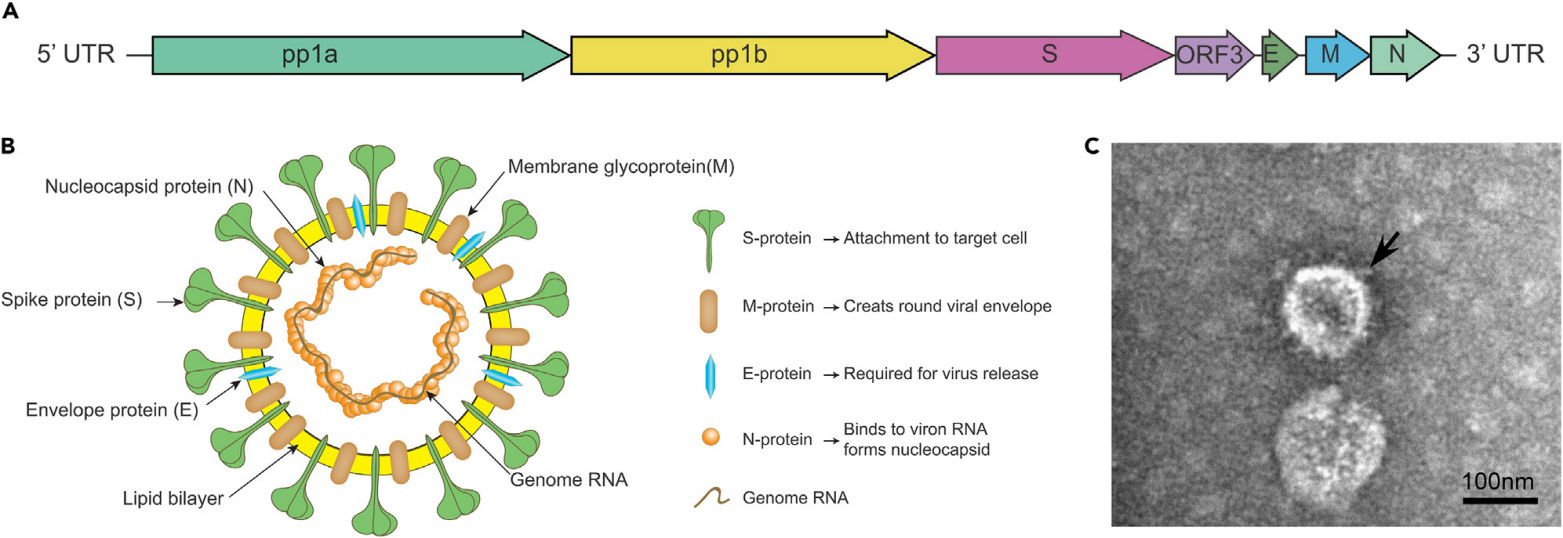
PEDV genome organization and viral structure (A) PEDV genome structure shows the position of the accessory protein and viral particle structure diagram. PEDV genome is divided into two sections, with the 5′ two-thirds containing two large, overlapping open reading frames (i.e., ORF1a and ORF1b). These ORFs encode two long polyprotein precursors, pp1a and pp1ab. The remaining 3’ third of the genome encodes four structural proteins, spike (S), envelope (E), membrane (M), and nucleocapsid (N), with an only accessory protein, ORF3. These proteins have distinct functions and each of them plays an essential role in the life cycle of the virus. (B) Schematic diagram of a PEDV virion is shown. Structural proteins of spike (S), envelope (E), and membrane (M) proteins are embedded in the lipid bilayer envelope. The viral genomic RNA (labeled with a gray line) is assembled with the nucleocapsid (N) protein, which is enclosed by the lipid bilayer membrane. The ORF3 accessory protein is not displayed in the virion structure because it is not a structural virion component at the current cognitive level. (C) Transmission electron microscopes (TEM) images of purified PEDV virions. The PEDV particles were negatively stained, and a virus particle is shown by an arrow. Crown-shaped spikes are visible. Scale bar: 100 nm. Magnification, ×,110,000.

Porcine epidemic diarrhea virus (PEDV), belonging to the genus *Alphacoronavirus* in the family *Coronaviridae* of the order *Nidovirales*, causes devastating enteric disease and high mortality in neonatal piglets.^1^ It is an enveloped virus with an approximately 28 kb positive-sense, single-stranded RNA genome that is 5’ capped and 3’ polyadenylated.^1^^,^^26^ The organization of the PEDV genome is typical of CoVs, with the gene order 5’UTR-ORF1a-ORF1b-S-ORF3-E-M-N-3’UTR (Figure 2A).^27^ The two polyproteins (pp1a and pp1ab) are encoded by the PEDV genome’s first two-thirds, and the four structural proteins (spike protein, S; envelope protein, E; membrane protein, M; and nucleocapsid protein, N) and one accessory protein (i.e., ORF3) are encoded by the remaining one-third of the genome (Figure 2A).

PEDV particle is a membrane-enveloped virion with an average diameter of about 95–190 nm,^1^ which is coated in a double-layer membrane with a characteristic “crown-like” structure on its surface (Figure 2B and 2C). The S protein is a type I membrane glycoprotein composed of S1 and S2 external domains and constitutes the characteristic spikes on the outside of the virion, facilitating viral attachment to target cells and producing-neutralizing antibodies.^28^ The E protein upregulates interleukin-8 expression, causes endoplasmic reticulum (ER) stress in the host cell, is proposed to possess ion channel activity, and participates in virion assembly.^29^^,^^30^ The thick lipoprotein virion envelope is made of the M protein, which is crucial for connecting the viral nucleocapsid and envelope.^31^ The N protein forms the viral core, participates in the transcription of the viral genome, binds to viral RNA, and forms the main component of ribonucleoprotein (RNP).^32^ The only accessory protein, ORF3, promotes virus proliferation, encodes an ion channel important for viral fitness, and plays an important role in several cellular processes.^33^^,^^34^^,^^35^^,^^36^ Although many research groups are currently studying ORF3 protein, numerous unknown properties of ORF3 protein still need to be elucidated.

## ORF3 is a multifunctional protein

The accessory proteins of various CoVs are involved in viral pathogenesis and virulence.^20^^,^^37^^,^^38^^,^^39^ In cell culture settings, they are not necessary for viral replication but may be crucial for controlling the host immune response, which could support viral stability and/or pathogenicity *in vivo*.^19^^,^^40^ For instance, even though the PEDV *ORF3* gene is often lost during the extensive cell passaging of the virus,^41^ its absence was related to diminished virulence.^42^ However, conflicting results of PEDV field strains with a naturally occurring truncated *ORF3* gene were recently found in China, and this natural deletion at the *ORF3* gene was confirmed to be highly virulent with severe diarrhea and high mortality to suckling piglets,^23^ which indicates that ORF3 protein plays a mysterious role in PEDV pathogenicity.

The *ORF3* gene has a length of 675 bp and encodes a protein of 224 amino acids. When PEDV is adapted to growth in cell culture, the protein is vulnerable to deletion or mutation.^41^^,^^43^ Studies showed that the deletion or variation of the *ORF3* gene might be related to the virulence and cell adaptation of the virus.^24^^,^^41^ During the continuous passage of PEDV *in vitro*, a 51-nucleotide (nt) loss in the *ORF3* gene is present in cell-adapted PEDV strains such as KPED-9 and P-5V, and this deletion is thought to be the primary cause of the PEDV strains’ decreased pathogenicity.^44^ Similarly, the attenuated DR13 and CV777 PEDV strain has also been found to have a 49-nt deletion in the *ORF3* gene, which causes an early termination of translation and a reading frameshift.^33^ Therefore, the *ORF3* gene’s ability to distinguish between cell-adapted and wild-type viruses enables it to be a valuable tool for epidemiological research of PEDV infections.^41^^,^^45^

Among the accessory proteins of all CoVs, PEDV ORF3 and SARS-CoV ORF3a share many similarities regarding protein structure and function. For instance, it has been demonstrated that SARS-CoV ORF3a is an ion channel protein and facilitates virus release.^46^ PEDV ORF3 protein is functionally analogous to SARS-CoV ORF3a and functions as a viroporin to control the generation of infectious progeny viruses.^33^^,^^46^^,^^47^ Besides its roles in affecting virus virulence and the cellular adaption phenotype with the truncated feature, ORF3 protein also plays multifaceted roles *in vitro*. Accumulating evidences suggest that ORF3 plays an essential role in numerous cellular processes. Ye et al. showed that PEDV ORF3 protein prolongs cellular S-phase and contributes to vesicle formation.^36^ Zou and co-workers proved that PEDV ORF3 protein triggers ER stress by upregulating GRP78 protein expression and activating the pERK-eIF2α signaling pathway,^34^ which further leads to autophagy in infected cells. Studies on the effect of ORF3 protein on apoptosis have also been reported. Chen et al. demonstrated that PEDV ORF3 protein could not induce apoptosis by transient transfection of ORF3 expressing-plasmid.^48^ However, by using artificially rescued PEDV recombinant virus strains carrying full-length *ORF3* gene (rPEDV-ORF3^wt^, rPEDV-ORF3^CV777,^ and rPEDV-ORF3^NY^) and the recombinant virus strains without *ORF3* gene (rPEDV-ΔORF3), our group found that ORF3 protein can delay the cytopathic effect (CPE) formation and significantly inhibit apoptosis induced by PEDV.^35^ More recently, Jiang et al. reported that the plaque size and syncytia phenotypes in the ORF3-null reconstituted PEDV were larger than those of wild-type ancestor. In addition, ORF3-null virus has a relatively faster growth phenotype,^49^ suggesting that ORF3 is dispensable for PEDV propagation *in vitro* with a potential role of ORF3 in PEDV cytopathology.

Furthermore, it has been found that ORF3 protein could interact with the PEDV spike protein during the virus infection,^50^ which indicates the possibility that ORF3 protein may assist S protein binding to cell receptors, thus promoting virus infection of cells. Wu and colleagues proved that PEDV ORF3 protein inhibited cellular proinflammatory cytokines (IL-6 and IL-8) production through NF-κB p65 pathway blockage, which provided a fresh perspective into PEDV ORF3 protein’s involvement in the immune evasion strategy.^51^ Kaewborisuth et al. found that ORF3 protein could not only downregulate the IFN-β promoter activation and suppressing poly (I:C) mediated type I IFN production and induction, but also upregulate IKBKB-mediated NF-κB promoter activity.^52^ It should be noted that cellular apoptosis played an essential role in virus-host interaction and innate immunity, closely related to efficient virus replication and pathogenesis.^53^^,^^54^ It is unknown whether ORF3 protein can highjack the key participating protein in innate immune response, such as the IFN-signaling pathway, and counteract the increased innate immune response, thus reducing type I IFN production. A summary of the ORF3 functions based on current knowledge is shown in Table 1. Altogether, PEDV ORF3 protein is a multifunctional protein that participates in numerous cellular processes and may be essential in virus infection, packaging, release, virus-host interaction, and maintaining normal host immunity.

**Table 1:** Subcellular localization and functions of PEDV accessory protein

Strain name	Features and functions	Reference
PEDV_AVCT12_	Suppress cell-adapted PEDV replication in cultured cells; Inhibit PEDV rescue in a reverse genetics system; Inhibits the manipulation of the viral genome and PEDV rescue; Inhibits recovery of PRRSV, but not influenza virus.	(Jengarn et al.^55^; Wongthida et al*.*^43^)
icPEDV	Deletion of ORF3 causes lower diarrhea scores and partial attenuation in pigs.	(Beall et al*.*^42^)
CV777	Consists of four putative transmembrane domains, functions as a potassium ion channel in both *Xenopus laevis oocytes* and yeast; Promotes viral replication of wild-type PEDV.	(Wang et al*.*^33^)
CH/YNKM-8/2013 (Field strain)	Facilitates the formation of double-membrane vesicles (DMVs); Promotes the proliferation of attenuated PEDV; Regulates the cell cycle progression by prolonging the S phase.	(Ye et al*.*^36^)
rPEDV	Contributes to virus proliferation and cell viability; Inhibits early cell apoptosis by suppressing Caspase-3 cleavage.	(Si et al*.*^35^)
Eukaryotic expressed	Causes ER stress response and promotes autophagy; Up-regulations of the GRP78 and activation of PERK-eIF2α signaling pathway.	(Zou et al*.*^34^)
PEDV_AV12_	Interacts with S protein during PEDV replication; Contributes to PEDV replication.	(Kaewborisuth et al.^50^)
DR13, YN144	Early termination of ORF3 could be the attenuation and cell adaptation marker of the PEDV.	(Song et al.^56^; Chen et al.^57^)
Eukaryotic expressed	Inhibits cellular proinflammatory cytokines IL-6 and IL-8 production; Blocks the NF-κB p65 activation and expression; Disrupts nuclear factor p65 phosphorylation and nuclear translocation.	(Wu et al.^51^)
PEDV_AV12_	Interacts with the IκB kinase β (IKBKB), resulting in the up-regulation of the IKBKB-mediated NF-κB promoter activity, down-regulation of the IKBKB-mediated IFN-β promoter, and IFN-β mRNA expression.	(Kaewborisuth et al.^52^)
rPEDV	Transports through the exocytic pathway; Not a structural virion component and constitutive protein of PEDV.	(Si et al.^58^)
PEDV_AV12_	Interacts with cellular VPS36 protein and associates with PEDV replication.	(Kaewborisuth et al.^59^)
	Induced autophagy in IPEC-J2.	
Eukaryotic expressed	Functions as the key inducers of PEDV-induced autophagy.	(Lin et al.^60^)
HN2021	Large deletion is associated with severe diarrhea and high mortality.	(Zhang et al.^23^)
NJ strain	Used as the target for the detection of classical PEDV strains.	(Liu et al.^61^)
Eukaryotic expressed	As an IFN suppressive protein, it inhibits type I and III IFN production.	(Zhang et al.^62^; Zhang et al.^63^)

**Note**: rPEDV means recombinant PEDV; ca-PEDV means cell culture adapted PEDV; icPEDV means infectious-clone-derived PEDV; Functions shown in bold is our observation.

## The Golgi-resident phenotype of ORF3 accessory protein and the supposed mechanism

The ORF3 protein was initially shown in the cytoplasm,^36^^,^^59^ specifically in the perinuclear area^50^ and the Golgi apparatus.^34^^,^^50^^,^^58^ These results suggest that ORF3 can relate to particular cellular pathways that might regulate viral replication and pathogenesis. Subsequent plasmids transfection and PEDV infection experiments showed that the wild-type ORF3 protein aggregated in Golgi and was partially expressed on the cell membrane, it was obviously a Golgi resident protein.^58^ However, the truncated ORF3 protein of the cell-adaptation strain DR13 had an altered intracellular localization, which presented only in the ER and could not be expressed on the cell membrane owing to a large amino acid deletion at its C-terminus.^58^ We further screened the amino acid motif that determines its subcellular localization and found that the ^170^YLAI^173^ motif (amino acids 170-173) determines its Golgi/ER retention and plays a decisive role in its cell membrane translocation.^58^ These findings raise an important issue: the involved mechanisms responsible for the Golgi retention and the cell surface expression of ORF3 protein, which need to be elucidated in greater detail.

Up to now, there are three possible mechanisms reasonable for the proteins retained in the Golgi apparatus: (1) Hydrophobic matching hypothesis: this hypothesis proposes that the protein containing a shorter transmembrane region will reside in a thinner membrane region, whereas the protein with a longer transmembrane region will be located in a thicker region.^64^ In other words, the transmembrane region affects the subcellular localization of the protein. At present, this hypothesis has been confirmed: on the one hand, the Golgi retention profile of yeast proteins (such as sed5) and SARS-CoV proteins (such as ORF7b) depend on the length of their transmembrane region.^65^^,^^66^ On the other hand, shortening the transmembrane region of VSV-G leads to a slower export rate out of the Golgi apparatus. Thus, the long transmembrane region contributes to the efficient export of secretory proteins;^67^ (2) Phase separation theory: phase separation is the process by which biomolecules (e.g., proteins and/or nucleic acids) are concentrated into a separate phase forming liquid droplets.^68^ Signal transduction in nerve cells is processed by phase separation, and synapses are the connection points where information transfer occurs between neurons and contain a compartment with a highly aggregated protein layer, the postsynaptic density (PSD), which is responsible for the processing and transmission of brain signals.^69^ It has been proved that PSD-95, a protein expressed in high abundance in PSD, can undergo phase separation with SynGAP at higher concentrations *in vitro*, which enhances protein plasticity and allows the protein to undergo phase changes in a short period, enabling an ultra-fast release of neurotransmitters and thus facilitating neural signaling.^70^ Phase separation also drives cargo protein sorting in chloroplasts,^71^ promotes cargo proteins translocated to vesicles, and facilitates protein sorting and transporting; (3) Kin-recognition model: the “kin-recognition model” was the first model proposed for the residency of glycosyltransferases in the Golgi.^72^ This model suggests that different transmembrane regions form so-called homodimers or heterodimers within the cell.^73^ The formed oligomers are too large to enter transport vesicles that sort cargo proteins exported from the Golgi, spatially preventing the loading of these proteins into the transport vesicles and therefore causing these oligomeric proteins to retain in the Golgi.^73^^,^^74^ Therefore, the retention of protein in Golgi is a complex process, and multiple factors are involved in the retention, among which the amino acid composition, aggregation state, and phase separation of protein are important influencing factors.

**Figure 3:**
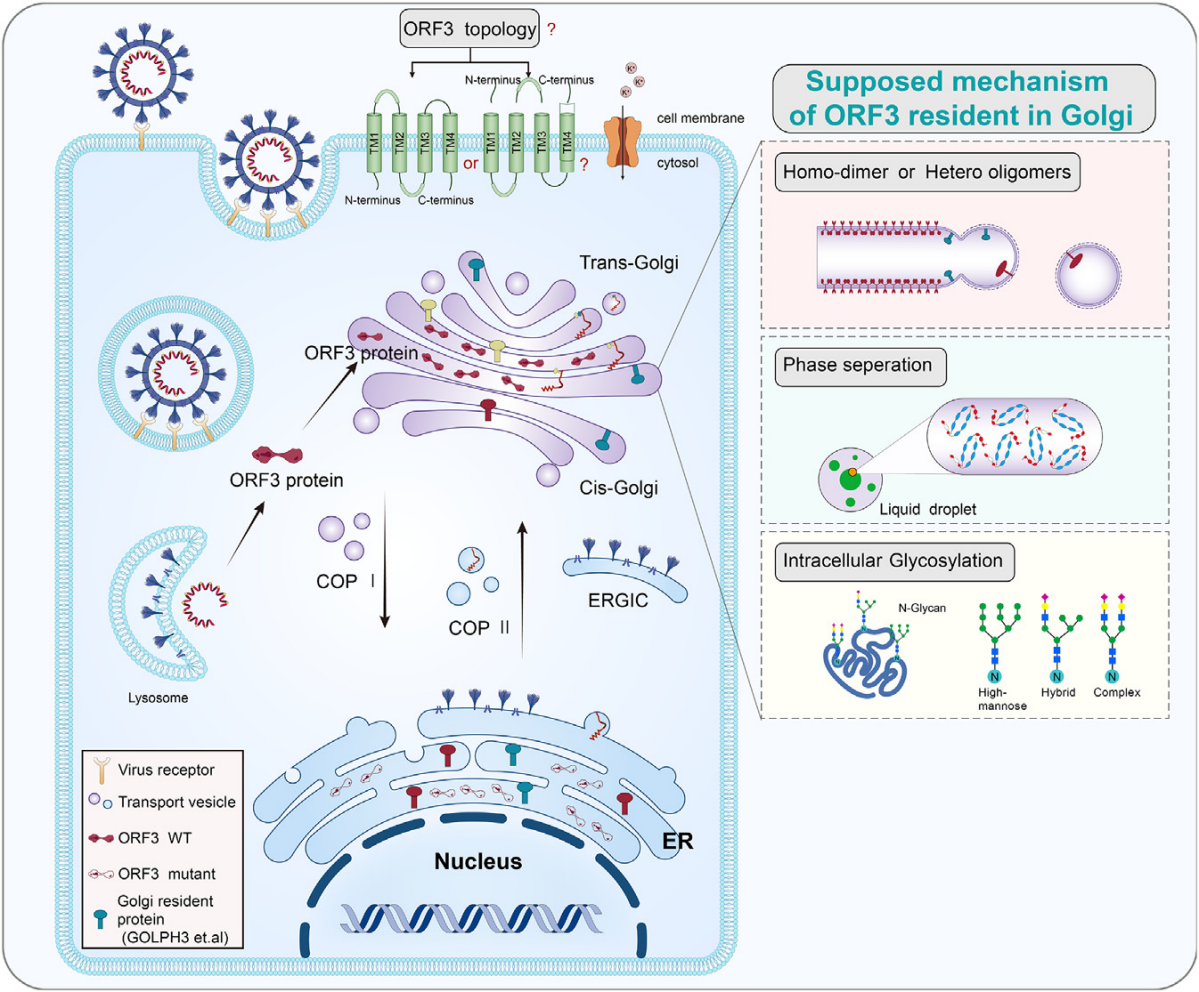
Working model for the roles of ORF3 protein in PEDV replication and the proposed retention mechanisms of ORF3 protein in Golgi apparatus After PEDV infection, the unique membrane topology of ORF3 protein affects the folding, post-translational modification and aggregation form of protein, which ultimately impacts its residency within the Golgi apparatus. On the one hand, through interprotein interaction, ORF3 protein recruits other Golgi resident proteins to form a highly complex protein interaction network; on the other hand, multi-spanning domain ORF3 proteins expressed in high abundance in cells undergo glycosylation modifications in the Golgi apparatus and promote phase separation or oligomerization through binding to membrane-anchored ion channels leading to the spontaneous formation of protein aggregates, which restrict the loading of ORF3 protein into transport vesicles and eventually lead to protein retention in the Golgi apparatus (This schematic diagram is the author’s own creation).

Currently, the related research on ORF3 protein is relatively limited. When considering the possible mechanisms responsible for the Golgi retention of ORF3 protein, the question of which of the above mechanisms is involved in the Golgi retention of ORF3 protein needs to be urgently revealed. Understanding this question will benefit the functional study of ORF3 protein. To the authors’ knowledge, one or more of the above mechanisms are likely involved in the phenotype of the ORF3 protein retention in the Golgi apparatus (Figure 3), and this work is one of the main topics currently ongoing in our laboratory.

## Membrane topology of ORF3 protein

**Figure 4:**
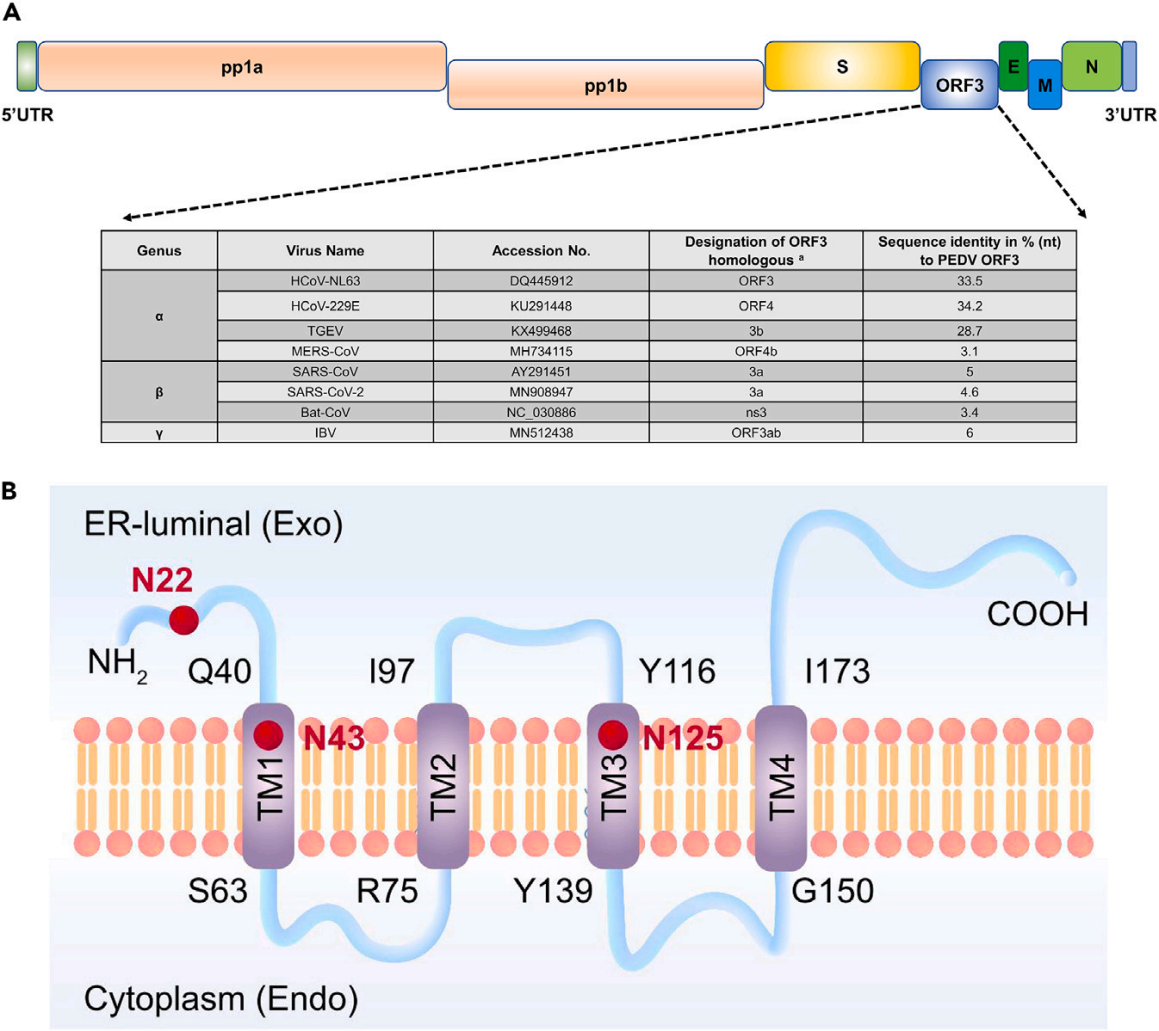
Characteristics of PEDV ORF3 and comparison to homologous genes in other coronaviruses (A) Localization of *ORF3* gene within the PEDV genome and comparison of nucleotide (nt) identity based on multiple sequence alignments with prototype strains of CoV groups alpha, beta, gamma. The sequence of ORF3 (protein_id = AFE85963) was analyzed using BLAST and MEGA-X software (version 10.1.8). Note that IBV ORF3a and 3b were fused to one ORF3ab. (B) Putative analysis on ORF3 protein membrane topology and glycosylation features. The putative four transmembrane (TM) domains, which including TM1 (Gln-40 to Ser-63), TM2 (Arg-75 to Ile-97), TM3 (Tyr-116 to Tyr-139), and TM4 (Gly-150 to Ile-173), are diagrammatically presented according to the previous study. Q, Glutamine (Gln); S, Serine (Ser); R, Arginine (Arg); I, Isoleucine (Ile); Y, Tyrosine (Tyr); G, Glycine (Gly); Predicted N-linked glycosylation sites are indicated by an “N” at the respective localizations with an index number identifying the amino acid position. No O-linked glycosylation sites are predicted.

Membrane topology affects the correct folding of proteins and impacts their function.^75^ Therefore, an important strategy for studying the coronavirus proteins in virus pathogenicity and infection is to clarify their subcellular location and topology. The coronavirus accessory protein has a complex membrane topology and is closely related to its cellular location and biological function. In most studies, SARS-CoV ORF3a has been used as an example to investigate the ORF3 function. The SARS-CoV ORF3a protein is predominantly located in the Golgi apparatus, with its N-terminus facing the extracellular matrix (Nt_lum_) and its C-terminus facing the cytoplasm (Ct_cyt_).^76^^,^^77^ It is a triple-spanning transmembrane (TM) protein that resembles the M protein in topology.^78^ In addition, it can form tetramers and potassium channels, regulate virus release,^46^^,^^79^ and is connected to a pro-apoptotic function.^47^ Similarly, a triple-spanning membrane topology structure was also found in SARS-CoV-2 ORF3a protein that forms cation channels in lipid nanodiscs and showed dimers and tetramers under cryoelectronic microscopy.^80^ Furthermore, TM1-TM2 and TM2-TM3 were connected by intracellular and extracellular short linkers, respectively.^80^ Between the S and E genes, the hCoV-NL63 genome encodes the ORF3 protein (Figure 4A), which is 225 aa long and has three putative transmembrane domains (TMDs) at aa positions 39-61, 70-92, and 97-116, respectively.^81^ Topology studies indicate that its N-terminal domain (NTD) is located in the extracellular region, whereas the C-terminal domain (CTD) is located in the intracellular region.^81^

The CoVs accessory protein is mostly found in intracellular membranes between the ER and Golgi compartments,^82^^,^^83^ where it plays important roles in the intracellular trafficking of the viral proteins and virus adsorption, invasion, packaging, budding, and release stages through an unidentified mechanism. In the case of PEDV accessory protein, it has been demonstrated that ORF3 protein is essentially accumulated in the Golgi area of the cell, similarly in infected and transfected cells.^58^ According to bioinformatic predictions, the PEDV ORF3 protein is a viroporin with four transmembrane domains and a potassium ion channel protein that controls virus release (Figure 4B).^33^ Results from our group showed that PEDV ORF3 protein is a Golgi resident protein, which diffusely aggregates around the nucleus.^58^ Although ORF3 protein promotes viral replication,^35^ it does not incorporate into virions purified by sucrose gradient ultracentrifugation.^58^ The above results indicated that the membrane topology of coronavirus proteins is closely related to their functions, and clarifying the protein’s topology can help to understand their functions in depth. Therefore, the defined membrane protein topology of PEDV ORF3 protein may provide a useful framework to understand its interaction with other viral and host components and contribute to establishing the basis to tackle the pathogenesis of PEDV. Although the ORF3 protein is currently predicted to be a multi-spanning transmembrane protein and confirmed to localize in the Golgi apparatus, its specific intracellular and extracellular regions and membrane topology in eukaryotes have not been demonstrated experimentally. Therefore, it’s urgent to clearly determine the specific transmembrane domains of PEDV ORF3 protein in eukaryotic cells and, specifically, to verify which domain is involved in the function of ORF3 protein.

## Potential intrinsic disordered property of ORF3 protein and the possibility of liquid-liquid phase separation (LLPS)

Intrinsically disordered proteins (IDPs), which are encoded by intrinsically disordered regions (IDRs) and earned the name “Dancing Proteins”, are a class of functional proteins lacking a fixed or organized three-dimensional structure,^84^^,^^85^ typically in the absence of its macromolecular interaction partners, playing crucial roles in the mechanism of virus pathogenesis, cell regulation and host signaling pathways.^86^^,^^87^^,^^88^^,^^89^ Overall, IDPs are characterized by their polypeptide segments, limited hydrophobic amino acids, and ability to facilitate various biological processes via mechanisms distinct from their structured counterparts.^90^ IDPs differ from structured proteins in many ways and tend to have distinctive functions, structure, sequence, interactions, evolution, and regulation.^89^^,^^91^

As obligate parasites, viruses achieve their infectious cycles through the recruitment of various host cellular components such as host proteins, nucleic acids, biological membranes, energy and metabolic machinery.^92^ Consequently, viral proteins frequently have multifunctional behaviors and engage in complex interactions with host ligands. During this process, IDRs could be quite helpful for viruses by ensuring binding diversity. Furthermore, IDRs endow viral proteins with considerable flexibility, whether completely disordered or partially, enabling them to quickly adapt to changing cellular conditions, survive in host immune surveillance environments, and antagonize the host’s defensive mechanism.^93^

It has been reported that IDPs are associated with many human diseases not limited to cancers, Down’s syndrome, Alzheimer’s disease, variants of Alzheimer’s disease, Parkinson disease, and prion disease.^94^^,^^95^ In addition, IDPs are also composed of various multifunctional proteins from different viral families, mainly concentrated in RNA viruses.^96^^,^^97^^,^^98^^,^^99^^,^^100^^,^^101^^,^^102^ For example, the SARS-CoV-2 N protein contains three dynamic disordered regions and undergoes liquid-liquid phase separation (LLPS) when mixed with RNA.^103^^,^^104^ IDRs are the main factor driving the formation of LLPS during this process.^89^^,^^96^^,^^105^ Furthermore, because they give viral proteins the ability to quickly and promiscuously bind to host proteins, the disordered regions of the SARS-CoV-2 N protein are typically linked to viral infectivity and pathogenicity.^98^ A recent study further proved that more than 90 disordered regions were found in SARS-CoV-2 spike protein, and the disordered regions in Omicron variants exhibit disorder-to-order transition when compared to its ancestral and the Delta variant strain, all of which indicated that the viral infectivity and pathogenicity were closely related to the IDRs of viral proteins.^106^ Although several therapies have been investigated, effective antiviral medications are now hard to come by. Therefore, a different approach for logical antiviral drug design could target the IDPs/IDRs in the SARS-CoV-2 genome.

**Figure 5:**
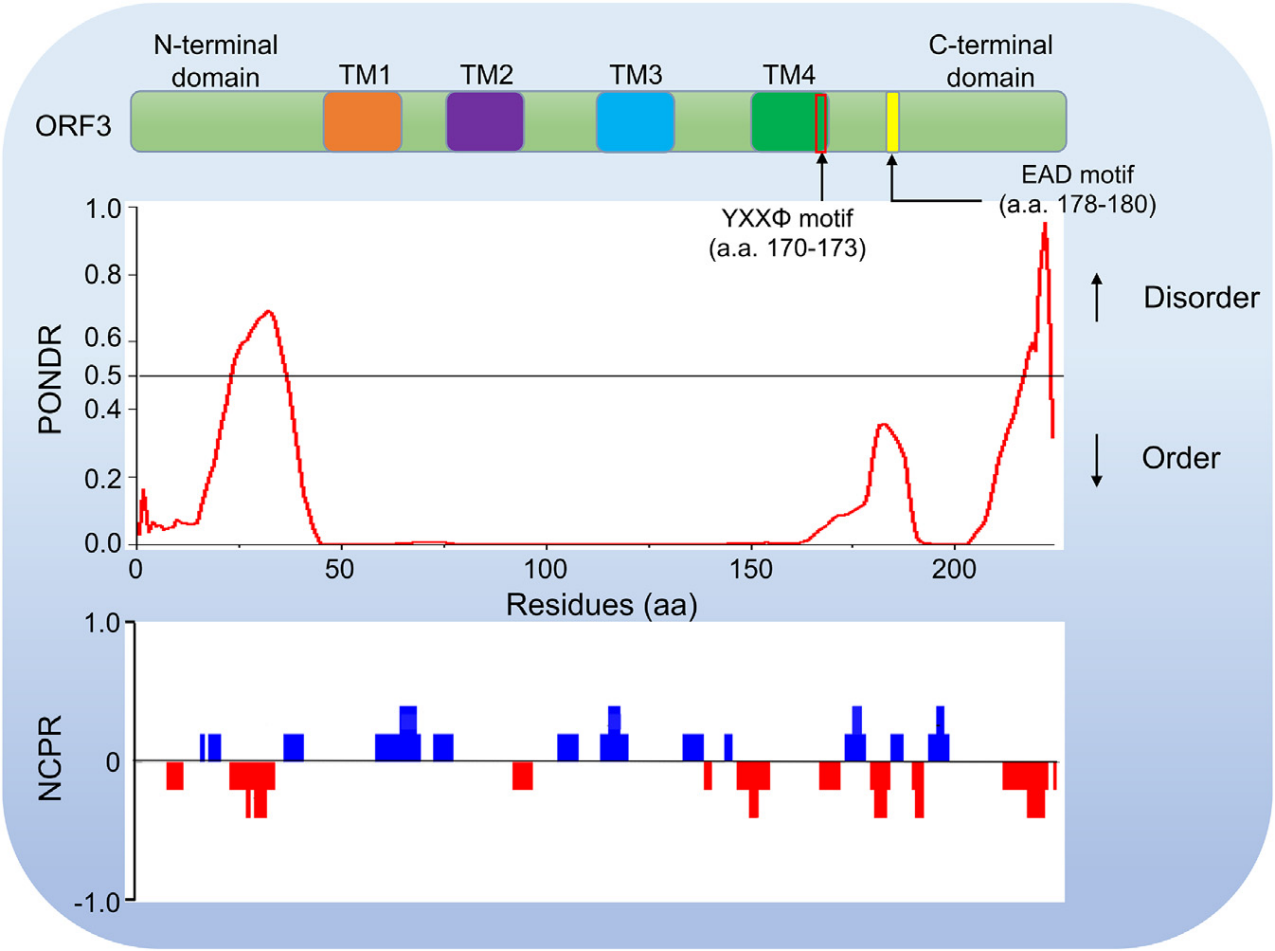
PEDV ORF3 protein domains aligned with results of PONDR (predictor of natural disordered regions) and NCPR (net charge per residue; 5-amino acid window) analyses A schematic representation of the domain structure and overall topology is shown on the top. The PONDR is applied to analyze the intrinsic disorder probability, and NCPR is employed to measure the net charge per residue. For the intrinsically disordered region prediction, residues with anticipated disorder scores of more than 0.5 are regarded as inherently disordered, whereas residues with expected disorder values between 0.2 and 0.5 are considered flexible.

As mentioned above and in other places, the disordered regions and the resulted LLPS property in viral proteins are closely related to the infectivity and pathogenicity of the virus.^88^^,^^107^^,^^108^^,^^109^ In light of PEDV, ORF3 protein is an important protein affecting virus virulence. Thus it is of certain interest to know whether the IDRs exist in the ORF3 protein coding sequence. According to current knowledge, ORF3 protein has three major domains: an N-terminal domain, a central segment predicted to undergo four times transmembrane, and a C-terminal domain (Figure 5). To test the phase separation ability of the ORF3 protein, we analyzed the sequence of the ORF3 protein using the PONDR (predictor of naturally disordered regions) tool.^110^ The results showed that there were two IDRs, residues 24-36 and 217-223 (Table 2), in ORF3 protein-coding sequences (Figure 5), indicating that the ORF3 protein has the potential possibility to undergo LLPS. Taken together, IDPs are related to human diseases and exist in various viral proteins, including ORF3. Therefore, the IDRs in the PEDV ORF3 protein coding sequence reminds us that we should strengthen the related research about the relationship between IDRs and virus virulence or pathogenicity, which will benefit us in understanding further the new function of ORF3 protein in great detail.

**Table 2:** Intrinsically disordered prediction of PEDV ORF3 protein using PONDR VLXT

Strain name/residues	Number residues disordered	Overall percent disordered	Predicted intrinsically disordered regions (IDRs)	Number of IDRs
DR13/224 aa^a^	20	8.93	[24]-[36]	2
			[217]-[223]	

a **Note**: aa means amino acid. The GenBank accession number of the DR13 protein sequence is ABS89138.

## Can ORF3 protein undergo glycosylation?

Protein glycosylation is an essential and highly conserved post-translational modification (PTM) reaction responsible for various key biological processes, including protein folding, oligomerization, sorting, transport, and stability.^111^^,^^112^^,^^113^ During those processes, glycans can demonstrate significant interactions between glycans and proteins and incorporate them into the protein fold to stabilize the protein, for example, through interacting with surface hydrophobic residues and increasing the solubility of proteins. In addition, glycans can also promote the folding of glycoproteins via influencing chaperone contacts during production.^114^ Generally, N-linked glycosylation and O-linked glycosylation are the two main kinds of protein glycosylation. When an oligosaccharide (glycan) is attached to the nitrogen atom of an asparagine residue, this process is known as N-linked glycosylation. This process typically occurs on asparagine residues in Asn-X-Ser/Thr sequons (asparagine residue in the-N-X-T/S- motif), where X can be any amino acid other than proline. O-linked glycosylation is the process by which a sugar molecule is joined to a protein’s serine (Ser) or threonine (Thr) oxygen atom. O-glycosylation affects the regulation and stability of proteins in the ER or Golgi apparatus.^115^ Although there are two other glycosylation types, phospho-glycosylation and C-linked glycosylation, they are rarely reported to be involved in the functional regulation of viral proteins.^116^

As obligate parasites, viruses employ host-cell machinery to glycosylate their own proteins during viral infection and proliferation, which plays an important role in virus-host interaction and host immune response.^117^^,^^118^ For example, the envelope proteins of various human pathogens, including the influenza virus hemagglutinin glycoprotein (HA),^119^ Dengue virus (DENV) envelope glycoprotein (Env),^120^ human immunodeficiency virus-1 (HIV-1) Env,^121^ Zika envelope (E) glycoprotein,^122^^,^^123^^,^^124^ Japanese encephalitis virus (JEV) premembrane (prM) glycoprotein,^125^ West Nile virus (WNV) prM and E glycoprotein,^126^ Ebola virus (EBOV) glycoprotein (GP),^127^ and Flavivirus Env^128^ are extensively glycosylated. Glycosylation on viruses, however, is not exclusive to the viral genus mentioned above. Many coronavirus proteins display glycans and undergo glycosylation which is essential for their specific functions, such as the spike and envelope glycoprotein (S and E) of SARS-CoV-2,^129^^,^^130^^,^^131^^,^^132^ the spike glycoprotein (S) of PEDV,^133^ the membrane protein (M) of infectious bronchitis virus (IBV)^134^ and SARS-CoV,^135^^,^^136^ and this is also the case in multitudinous coronavirus accessory proteins, which include 3a of SARS-CoV, ORF3 of human pathogenic coronavirus NL63 (hCoV-NL63),^81^ 7b of feline coronaviruses (FCoVs),^137^ and the secreted ORF8 of SARS-CoV-2.^138^ In addition, the glycosylation machinery in the ER-Golgi system of host cells can be hijacked by viruses to help escape the host’s immune surveillance and shield them from antibody recognition, which is crucial for multiple facets of viral pathogenesis.^114^^,^^139^ This is true for coronavirus, for example, it has been reported that targeting or inhibiting the protein N-glycosylation blocks SARS-CoV-2 infection.^140^^,^^141^^,^^142^

The active site of oligosaccharyltransferase (OST), a membrane protein complex that chooses the Asn-X-Ser/Thr consensus sequence on polypeptide chains and creates the N-glycosidic linkage between the side-chain amide of asparagine and the oligosaccharide, is only found in the lumen of the ER in eukaryotic cells.^143^ Therefore, neither the membrane nor the cytosol experience N-linked glycosylation.^130^ According to *in silico* analysis, although several amino acid mutations were found through the ORF3 multiple sequence alignment (Figure S1A), the ORF3 protein indeed contains three potential and conserved N-linked glycosylation motifs, asparagine-X-serine/threonine (X is any amino acid except proline), or three putative N-glycosylation sites at positions N22, N43 and N125 (Figure 4B), of which probably only the first is used because the sites at positions 43 and 125 are located inside the predicted transmembrane domains, whereas only the N22 is located in the ER lumen (Figures 4B and S1B). Up to now, there has been no prediction or study on the PEDV ORF3 protein’s glycosylation. Therefore, it is urgently needed to experimentally verify whether the ORF3 protein undergoes N-glycosylation in mammalian cells and which of the predicted N-linked glycosylation motifs is authentic for ORF3 protein. Elucidating this issue will further reveal the biological functions of ORF3 proteins.

## Knowledge of functional motifs on ORF3 protein

The ER/Golgi intermediate compartment (ERGIC) or the Golgi compartment, where are the locations of CoV assembly and packaging, CoV accessory proteins were found to have similar localizations in these organelles.^144^ Some CoVs accessory proteins functioned as the constitutive component of virions and integrated into virus particles, such as ORF4a of human coronavirus 229E (HCoV-229E),^145^ 3a and 7b of SARS-CoV,^78^^,^^82^ and ORF3 of hCoV-NL63,^81^ which could explain the essential role that numerous accessory proteins play in viral infection. CoVs accessory proteins, including PEDV ORF3 protein, frequently contain YXXΦ and acidic motifs generally situated at the cytoplasmic domain (C-terminus), which determine the trafficking and turnover of many membrane-spanning proteins in the cell.^58^^,^^146^^,^^147^^,^^148^ Based on current published data, a summary of the YXXΦ and ExD (di-acidic) motifs found in some well-known transmembrane proteins and the C-terminus of PEDV ORF3 protein are summarized in Table 3.

**Table 3:** Comparison of YXXΦ and di-acidic motifs found in the well-known transmembrane proteins and the cytoplasmic domain of PEDV ORF3 protein

Protein	NCBI identifier (No.)	YXXΦ and di-acidic motifs	Reference
ORF3 ^parent DR13^	EU054929	TM-VAFVSSIDLYLAIRGRQ**EAD**LHLLRTV-COOH	(Park et al*.*^44^)
ORF3 ^Virulent DR13^	JQ023161	TM-VAFVSSIDLYLAIRGRQ**EAD**LHLLRTV-COOH	(Park et al.^149^)
ORF3 ^CV777^	AF353511	TM-VAFVSNIDLYLAIRGRQ**EAD**LHLLRTV-COOH	(Bridgen et al.^150^)
ORF3 ^OK10240-8^	MG334555	TM-VAFVSSIDLYLAIRGRQ**EAD**LQLLRTV-COOH	(Zhang et al.^151^)
ORF3 ^USA Oh851^	KJ399978	TM-VAFVSSIDLYLAIRGRQ**EAD**LQLLRTV-COOH	(Wang et al.^152^)
ORF3 ^USA PC22A^	KY499262	TM-VAFVSSIDLYLAIRGRQ**EAD**LQLLRTV-COOH	(Hou et al.^153^)
ORF3 ^China GD−1^	JX647847	TM-VAFVSSINLYLAIRGRQ**EAD**LHLLRTV-COOH	(Wei et al.^154^)
ORF3 ^HeN−MY−2015^	KU641647	TM-VAFVSSIDLYLAIRGRQ**EAD**LQLLRTV-COOH	(Wang et al.^155^)
VZV-GPE	P09259	TM-NQSMYYAGLPVD**DFE**DSESTDTEEE-COOH	(Olson and Grose,^156^)
VSV-G	P03522	TM-GIHLCIKLKHTKKRQIYT**DIE**MNRLGK-COOH	(Rose et al.^157^)
Human LAP	P11117	TM-LLTVLFRMQAQPPGYRHVA**DGE**DHA-COOH	(Chen et al.^158^)
Human LDLR	NM_000527	TM-LKNINSINFDNPVYQKTT**EDE**VHICHN-COOH	(Ference et al.^159^)
Human ASGPR1	P07306	TM-MTKEYQDLQHL**DNE**ESDHHQLRKGP-COOH	(Spiess and Lodish,^160^)
Mouse CD3 γ chain	P11942	TM-DKQTLLQNEQLYQPLK**DRE**YDQYSH-COOH	(Haks et al.^161^)
Mouse CD3 δ chain	P04235	TM-QALLKNEQLYQPLR**DRE**DTQYSRLG-COOH	(Van Den Elsen et al.^162^)
Human GLUT4	P14672	TM-HRTPSLLEQEVKPSTELEYLGPD**END**-COOH	(Wollscheid et al.^163^)
Mouse GLUT4	Q27994	TM-HRTPSLLEQEVKPSTELEYLGPD**EHD**-COOH	(Abe et al.^164^)

**Note**: The YXXΦ motif is underlined, and the acidic residues complying with the di-acidic [(D/E)X(E/D)] signal are boldly displayed. Sequences were retrieved from the NCBI database (https://www.ncbi.nlm.nih.gov/).

Those functional motifs were well studied to some extent and contributed to their intracellular transport and biological function. For example, SARS-CoV 3a protein, previously termed U274, comprised of 274 amino acids and three putative transmembrane domains, was found on the plasma membrane and perinuclear area. According to the analysis of the C-terminal domain of the 3a protein, two distinct sorting motifs, a YXXΦ (where X is any amino acid and is an amino acid with a bulky hydrophobic side chain) upstream of an ExD (di-acidic) motif, were found. The YXXΦ motif has been linked to dominating the viral protein’s intracellular localization in various host-cellular organelles,^165^^,^^166^^,^^167^ whereas the di-acidic motif is essential for efficient ER export.^168^ These two motifs’ juxtaposition seems crucial for transporting proteins to the plasma membrane.^169^ It was also proved that these motifs could play a significant role for SARS-CoV 3a protein to induce cellular apoptosis,^170^ and cell-cycle arrest,^76^ which was also detected in SARS-CoV-2 3a protein, was also linked to the induction of apoptosis during virus infection.^148^ Furthermore, previous and recent studies demonstrated that the occurrence of point mutations of the 3a proteins’ YXXΦ motif in SARS-CoV and SARS-CoV-2 showed a weakened pro-apoptotic phenotype,^47^^,^^148^ indicating the significant roles of these functional motifs on 3a’s pro-apoptotic function.

Similar motifs are also found in the ORF3 protein encoded by the PEDV genome.^58^ In our previous studies, we proved that the PEDV ORF3 protein possesses the YXXΦ and di-acidic motifs in its coding sequence. The YXXΦ motif (^170^YLAI^173^) located at the C-terminal domain was confirmed to be a key motif affecting the intracellular transport of ORF3 protein, and determining its expression on the cell membrane.^58^ Furthermore, ORF3 protein is closely related to PEDV-induced apoptosis.^35^ Therefore, it is interesting to investigate whether YXXΦ and di-acidic motifs on ORF3 protein are involved in regulating PEDV-induced apoptosis. If so, what are the similarities and differences of the regulatory mechanisms between the motifs on ORF3 and 3a protein? This work is currently ongoing in our laboratory. Though the identification and characterization of the functional motifs of PEDV ORF3 protein in regulating virus proliferation and apoptosis have not yet been revealed, ORF3 protein likely has a similar role in this regard because of its similar features (viroporin, same location between S and E, and the multi-spanning transmembrane peculiarity) to SARS-CoV and SARS-CoV-2 3a protein. Many researchers are becoming more interested in the crucial role of ORF3’s functional motifs in promoting PEDV pathogenicity and virus-host interaction because of their potential merits in antiviral targets and attenuated vaccine development. In addition, these changes were associated with virus growth and pathogenicity and whether those mutations impact PEDV field strains adaptation in cell culture is still not elucidated. Thus, those issues deserve to be explored in depth based on the current knowledge.

## Codon usage of ORF3 protein and its adaptive evolution phenotype

Synonymous codons are those codons that encode the same amino acid. Synonymous codon usage is not randomly used; some codons are utilized more frequently than others.^171^^,^^172^ This phenomenon is known as synonymous codon usage bias (CUB) in numerous organisms, including prokaryotes, eukaryotes, and viruses.^172^^,^^173^^,^^174^^,^^175^^,^^176^^,^^177^^,^^178^^,^^179^ Synonymous CUB is generally influenced by factors including mutational pressure, natural selection, translational efficiency, and mammalian genome compositional restrictions.^171^^,^^180^ A recent report shows that some novel influencing factors, including mRNA export, transcription, and immune evasion, can also dominate the viral codon usage,^173^ and the virus’s CUB serves as an important influencing factor in viral adaptation to the host and determines the viral host tropism.^181^^,^^182^^,^^183^

It is documented that the virus’s capacity to evade host immune surveillance, its ability to survive in extreme conditions, and its genetic evolution capacity are all significantly influenced by the codon usage between the virus and the host.^173^^,^^184^ Therefore, understanding viral codon usage can yield valuable knowledge about gene expression and regulation based on codon adoption, which can benefit the development of a new generation of vaccines that achieve a high level of viral antigen expression to induce long-lasting immunity.^185^ Thus, codon usage analysis is a powerful tool to elucidate the CUB of multiple organisms. Knowledge about the CUB’s profundity and potential influencing factors is critical for elucidating the viral evolution pattern and host adaptability. Thus far, few studies have investigated how animal viruses use synonymous codons. PEDV is an animal RNA virus that exhibits a rapid evolution rate since it first appeared. Previous studies on PEDV have mostly focused on infectivity and prevalence.^1^^,^^32^^,^^186^ However, there are limited studies on the CUB of PEDV, which is especially true for studying PEDV ORF3 protein’s CUB.

Chen et al. identified that PEDV has a rare codon usage pattern in its genome, indicating that the frequency of synonymous codon usage is dispersed throughout the PEDV genome.^187^ In addition, they discovered that PEDV’s codon usage pattern is shaped by two primary factors, mutational pressure and natural selection, with the latter playing a more significant impact. Furthermore, other factors, such as geographical distribution and the dinucleotide composition, were regarded as other potential influencing factors shaping the PEDV’s codon usage pattern to some degree. Two published results proved those conclusions, indicating a conservative and invariable codon usage preference in the PEDV genome.^183^^,^^188^ Furthermore, our group demonstrated that PEDV had evolved a mixture of antagonistic and coincident codon usage patterns relative to *Sus scrofa*, which promote its host adaptation and viral replicative fitness, and the PEDV genotype II strains show the highest amount of adaptation phenotype than other divergent clades.^183^

Though several studies have previously mentioned the CUB of the whole PEDV genome, research on the codon usage pattern of a particular gene within the PEDV genome is still very scarce. Up to now, only three single gene’s codon usage patterns have been reported among the serious genes of the PEDV genome. Cao et al. showed a modest CUB in PEDV S protein and further demonstrated that mutational pressure, not translational selection, was the primary influence on this bias. In addition, they also discovered that the S gene’s aromaticity and hydrophobicity slightly influenced the variance in this phenomenon.^189^ The PEDV ORF3 genome’s codon usage patterns were then thoroughly analyzed by Xu and colleagues, who discovered that the PEDV ORF3 genome includes the CUB and that the CUB is low.^190^ In addition, the codon usage pattern is influenced by two main factors: mutational pressure and natural selection, with the latter having a greater impact on the CUB. Moreover, it has been discovered that other elements, such as dinucleotide composition, hydrophobicity, and aromaticity, affect the diversity in codon usage across the PEDV *ORF3* genes.^190^ Sheikh et al. comprehensively investigated the genetic evolution among the N genes of several CoVs, including PEDV, and found the PEDV genomes have undergone rapid evolution, and the PEDV N gene’s mutation has had a significant impact on evolutionary selection.^191^

The above results demonstrated that although a single PEDV gene (S, N, or ORF3) and the complete PEDV genome have a similar low CUB, the codon usage pattern of a single gene cannot represent the whole PEDV genomes and vice versa. It is believed that knowledge about the codon usage patterns of the viral gene and the related influencing factors is beneficial for understanding virus evolution.^192^ Therefore, systematic genome analysis was needed to elucidate PEDV’s evolution mechanism and codon usage pattern. Considering the fatal harm of PEDV to pigs and the important role of the *ORF3* gene in virus pathogenicity, as well as the continuous emergence of naturally occurring truncated *ORF3* gene in clinically isolated PEDV strains,^23^^,^^24^ it is necessary to track and analyze the codon usage profile of PEDV *ORF3* gene in the process of virus evolution. The pertinent findings are valuable for vaccine devolvement strategies because they will offer crucial details on virus evolution, gene transcription, regulation, and protein expression, as well as more assistance in assessing a virus’s host adaptation and evolution.^191^

## The perspective of ORF3 biology to the field of coronaviruses in general

Given the various biological properties of ORF3 accessory protein described above, we believe that ORF3’s biology plays a potential role in the pathogenesis of PEDV, and they are ubiquitous among coronavirus accessory proteins, which are important “catalysts” for studying the pathogenesis of the coronavirus family. For example, our previous study showed that in the process of PEDV rescue by reverse genetics, deletion of the *ORF3* gene and replacement with the heterologous green fluorescent protein gene (GFP) resulted in the successful rescue of PEDV and proliferation of the offspring virus on cells,^35^^,^^58^^,^^193^ suggesting that the accessory proteins in the coronavirus genome can be deleted or replaced with a heterologous reporter gene, which indicates that it is of great importance for the development of current vaccines for SARS-CoV-2 and other CoVs, as well as for the study of their pathogenesis. Indeed, this phenomenon has been confirmed in the current development of the live-attenuated SARS-CoV-2 vaccine and other coronavirus vaccines.^43^^,^^49^^,^^193^^,^^194^^,^^195^^,^^196^^,^^197^ In our recent study, we also found that the PEDV accessory protein is a Golgi resident protein, and the two functional motifs (i.e., YXXΦ and ExD) play an important role in its retension.^58^ Furthermore, we found that protein membrane topology, intrinsically disordered regions, functional motifs, post-translational modification reactions (especially glycosylation) and codon usage patterns that affect the biological properties of the accessory proteins are also involved in the Golgi retention mechanism of ORF3 protein. Given that the ORF3 protein shares many similarities regarding the genomic structure and is functionally analogous to those of human and other animal coronavirus accessory proteins, we speculate that some biological properties of ORF3 protein are universal to other coronavirus accessory proteins, as it has been demonstrated that the accessory protein in PEDV and SARS-CoV-2 genome can be deleted and replaced with heterologous GFP and this manipulation does not affect virus recovery and vaccine development application.^49^^,^^194^^,^^196^ However, many aspects have not yet been reported in studies investigating the function and properties of human and other animal coronavirus accessory proteins. From this perspective, the PEDV ORF3 biology study may open up a new avenue for decoding of other coronavirus accessory proteins’ biological function. Therefore, through this study, we call for virologists to strengthen cooperation in this regard in the future worldwide, further confirm and extend the accessory proteins’ biological characteristics to the general coronavirus field.

## Concluding remarks and future perspectives

The coronavirus has been circulating in the world of veterinary medicine for a long time. Given the cross-species transmission potential of coronavirus, the continuous prevalence of coronavirus in animals poses a serious threat to human health. Therefore, to some extent, a consensus of One Health that emphasizes the relationship between humans, animals, and the environment and recognizes that human health and well-being are strongly influenced by the health of animals and their environment,^198^ eventually appears as an important concept and is acknowledged worldwide.

Coronavirus accessory proteins are one of the important factors affecting virus virulence, participating in multiple viral infection processes and contributing to pathogenicity. Some of them are thought to play an important role in reshaping the viral virulence and immune surveillance,^199^ but how they influence those viruses’ biological behaviors remains obscure. In the case of PEDV accessory protein, ORF3, although a recent mini study summarized its ability to modulate host responses, the role in viral replication, the contribution to virus pathogenicity, and host-virus interaction,^25^ there are still many covered spots that have not been stressed in this work, especially it failed to elaborate the biological characteristics of ORF3 protein, which is of great importance for the functional execution and viral pathogenesis. In this situation, here we focus on the research progress on unique characteristics of ORF3 accessory protein, that are important for them to play the biological role and emphasize several aspects that need to be elucidated extensively and urgently, for the purpose of reminding the virological community to pay more attention to the related research on all CoVs accessory proteins among the four genera including PEDV’s, especially the investigation on the biological characteristics of the proteins.

In recent years, although the research on PEDV ORF3 protein has made some progress, it is still limited compared with the research progress on structural proteins, and researchers still have a poor understanding of ORF3 protein’s biological characteristics. For example, the research on its membrane topology is still in the prediction stage, so far, the predicated membrane topology of ORF3 protein is not experimentally confirmed; even some results related to PEDV replication and its genome manipulation were conflicting among the available literature^35^^,^^42^^,^^55^; in addition, whether the functional motifs of ORF3 protein is involved in virus proliferation, viral pathogenicity, host innate immune response and the regulation of virus-induced apoptosis is still mysterious; more importantly, though we proposed the potential retention mechanism of ORF3 protein in Golgi apparatus based on the current research progress (Figure 3), its detailed mechanism needs to be confirmed by thorough experiments; furthermore, continuously genetic evolution monitoring of ORF3 variation in novel PEDV field strains is absolutely needed, as the genetic variation of *ORF3* gene is closely related to the pathogenicity and virulence of PEDV; finally yet importantly, because IDR is one of the main regulatory factors driving the occurrence of LLPS,^89^^,^^105^^,^^200^ it is necessary to determine whether ORF3 protein undergoes LLPS, and whether LLPS affects the PEDV’s kinetic behavior. In addition to the above-mentioned issues, it is still unknown whether ORF3 protein undergoes PTMs, such as glycosylation, phosphorylation, methylation, ubiquitylation, and palmitoylation, and how they regulate the virulence and pathogenicity of the virus during virus infection. Therefore, the gaps mentioned above should be investigated more deeply, and these posed research blind spots in the virological profiles of ORF3 will guide further studies to reveal its biological functions and benefit its counterparts in human coronavirus.

## Limitations of the study

Despite the current study concerning coronavirus accessory protein’s biology is investigated in great detail and discussed in various aspects, there are still some limitations that remain. First, given that this work takes the PEDV ORF3 protein as an example to emphasize the important role of coronavirus accessory protein biology in viral behavior and pathogenesis, it is unknown whether our conclusions would be applicable to a larger number of other accessory proteins located at the 3’-proximal genomic regions; in addition, although our published data and preliminary experimental results suggest the crucial role of accessory protein biology in the coronavirus life cycle, it is still necessary to continue studying and confirming the potential mechanisms involved in this process; furthermore, in this study, we focused on the similar biological characteristics of PEDV ORF3 accessory protein and several human coronavirus accessory proteins (e.g., SARS-CoV 3a protein, SARS-CoV-2 3a protein, and HCoV-NL63 ORF3 protein), which all located between the S and E gene loci. However, because most coronavirus genomes encode several accessory proteins located at different locations of the genome, there is an urgent need to elucidate the biological properties of coronavirus accessory proteins distributed beyond the S and E gene loci. These issues are major challenges waiting to be crossed by researchers. By improving our understanding of these proteins’' biology, we can develop more effective treatments and vaccines to control the spread of various emerging and re-emerging coronavirus diseases including COVID-19.
