# Supplementary material for: Coronavirus accessory protein ORF3 biology and its contribution to viral behavior and pathogenesis
Source: iScience. 2023 Feb 28;26(4):106280. doi: 10.1016/j.isci.2023.106280 (PMC9972675; doi:10.1016/j.isci.2023.106280)
Supplement: Document S1. Figures S1 [file mmc1.pdf]

iScience, Volume *26*

Supplemental information

Coronavirus accessory protein ORF3 biology and its contribution to viral behavior and pathogenesis

Fusheng Si, Shuai Song, Ruisong Yu, Zhen Li, Wenqiang Wei, and Chao Wu

Supplementary Figure 1

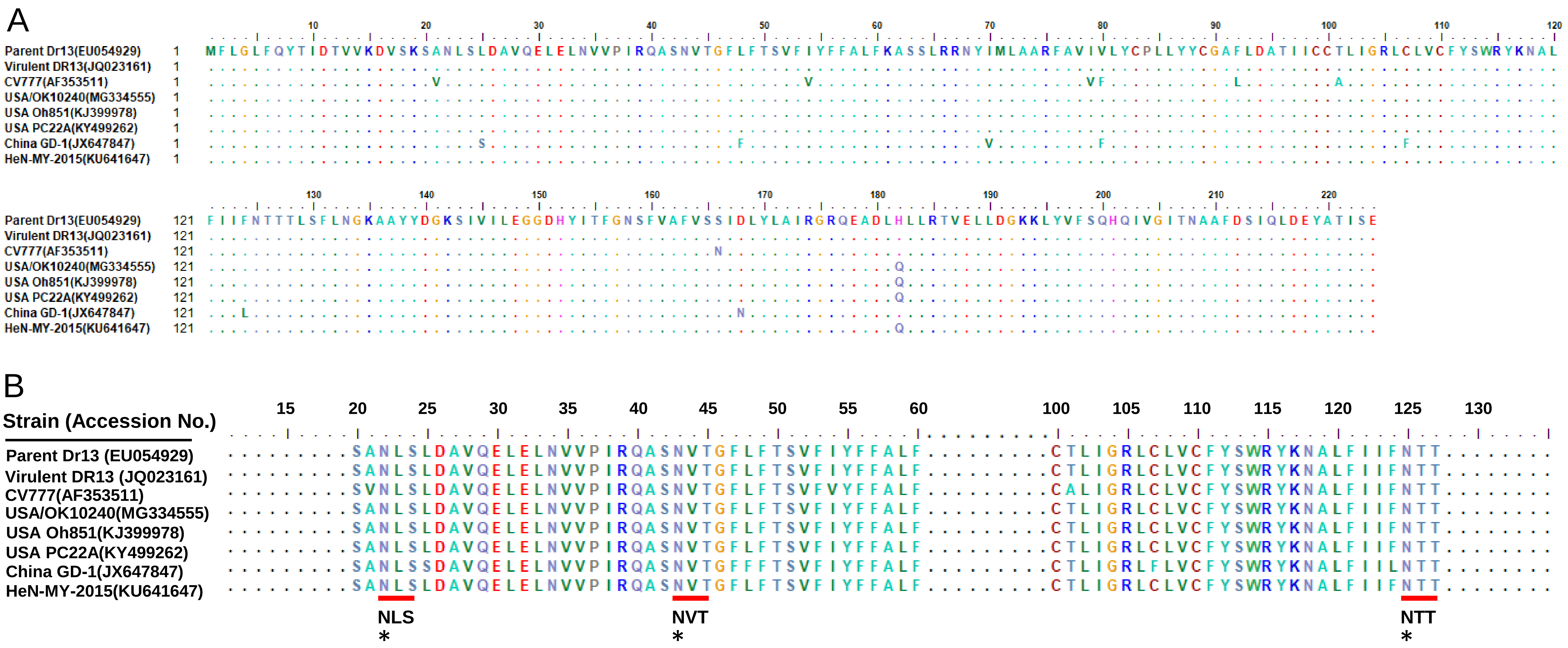

Supplementary Figure 1. Multiple sequence alignment and putative glycosylation features of ORF3 protein. Related to Figure 4.

(A) Multiple alignments of the full-length amino acid sequence of representative ORF3 protein isolated from different PEDV strains. The top row indicates the residue positions wherein the number indicates the interval of 10 amino acids, and the uppermost sequence (Parent Dr13, EU054929) is considered the reference sequence. GenBank accession numbers are presented on the left panel, and dots represent identical amino acids. Alignments and sequence analysis were performed using the BioEdit software package (version 7.0.9).

(B) Putative glycosylation sites on the ORF3 coding sequence. A comparison of amino acid sequences in the indicated region of ORF3 protein sequences was retrieved from the National Center for Biotechnology Information (NCBI). The putative asparagine residue in the -N-X-T/S- motif is underlined red, and an asterisk indicates the N-linked glycosylate site. Dots represent amino acid sequences not shown.
